# Exploring the Use of Non-Image-Based Ultrasound to Detect the Position of the Residual Femur within a Stump

**DOI:** 10.1371/journal.pone.0164583

**Published:** 2016-10-20

**Authors:** Sook-Yee Chong, Oliver Röhrle

**Affiliations:** 1 Institute of Applied Mechanics, University of Stuttgart, Stuttgart, Germany; 2 Biomechatronics System, Fraunhofer IPA, Stuttgart, Germany; Shanghai Jiao Tong University, CHINA

## Abstract

A satisfactorily fitted socket interacts dynamically with the stump in order to support body weight, transmit load effectively, enhance dynamic stability, and enable the control and stabilization of the residual limb. The internal dynamics occurring within a socket is important in determining optimal fit. Many measurement and imaging techniques, such as X-rays, have been utilized to investigate the movement of the residual femur within the stump during gait. However, due to associated health risks and costs, none of the current techniques have been extended to clinical prosthetics. The use of B-mode ultrasound has been suggested as a safe and cheap alternative, and has been utilized in previous studies to monitor the motion of the femur. However, the need to create a duplicate socket and time-consuming analysis of the images were obstacles to the system being applied clinically. This study aims to gauge the effectiveness of a non-image based ultrasound system. Here, we determined errors expected from the measurements. Accuracy errors of 2.9 mm to 8.4 mm and reproducibility measurements within a standard deviation of 3.9 mm are reported. We also estimated errors up to 14.4 mm in in-vivo measurements. We think there is potential in developing this technique, and we hope to reduce some technical difficulties such that it can, one day, be easily incorporated into prosthetic fitting.

## Introduction

A satisfactorily fitted socket interacts dynamically with the stump, in order to support body weight, transmit load effectively, enhance dynamic stability, and enable the control and stabilization of the residual limb. In addition, it has to remain comfortable and functional for the amputee. An important biomechanical consideration during a socket fitting is the interaction between the residual limb and the prosthetic socket. An understanding of both the internal biological structure of the residual limb, and the transmission of load from the skeleton to its surrounding tissues, would help determine and provide an optimal fit.

**Fig 1:**
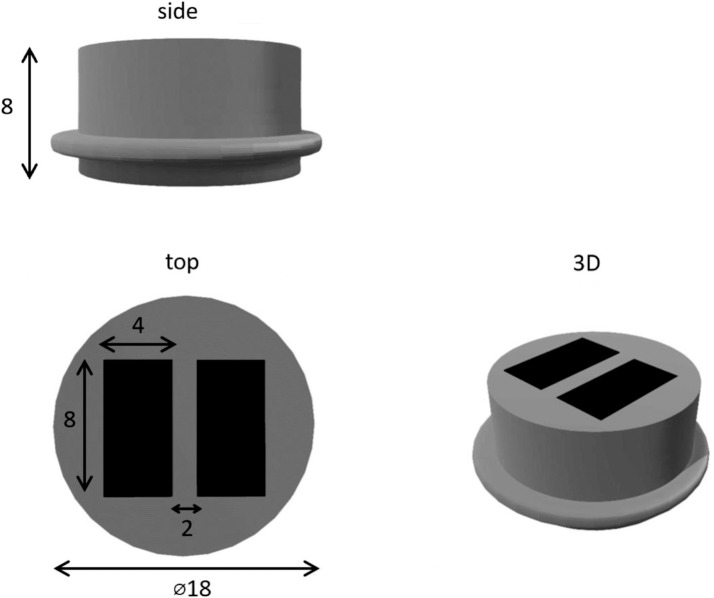
2 MHz transducer used in this study. All measurements are in mm.

In the 1940s, the quadrilateral socket was introduced for transfemoral amputees, as an effective design for the positioning of the ischium, and in controlling and stabilizing the femur during gait. However, by the 1960s, biomechanical problems were reported, such as amputees displaying the “lateral trunk leaning” gait. This is where an amputee has to lurch towards the amputated side during stance. Since the socket was too wide medial-laterally, too narrow anterior-posteriorly, and the ischium free to move about, the unsupported femur was angled towards abduction during weight bearing, resulting in high pressure occurring at the lateral distal and medial proximal regions [1,2] (For illustrations of the “lateral trunk leaning” gait, please refer to Figures 1 and 2 in Sabolich, 1985). To mitigate this problem, Long (1985) developed the ischial containment socket design so as to place the femur into an adduction position [1]. This position would allow better gait efficiency through the action of the gluteus medius [3]. Thus, the relationship between the position of the femur and the socket is an important criterion in determining the outcome during socket fitting.

Attempts to determine bone and/or soft tissue movements within the socket are not new. Previous studies have used roentgenologic measurements to measure the residual distance between the tibia and the socket at specific stride positions in a below-knee prosthesis [4–6]. Mean tibial movement in the anteroposterior direction was 2.2 cm, and the proximodistal distance was determined to be 2.25 cm [4] to 2.8 cm [6].

Studies using X-ray technique for the above-knee prostheses have mainly looked into adduction/abduction angles of the hip [1,7]. However, Erikson and James (1973) also reported femur movement as much as 2.3 cm from non-weight-bearing to full weight-bearing on the prosthesis, and the femur’s position being from 3.1 cm up to 11.3 cm away from the medial wall of the socket [8]. While X-rays can clearly identify bony landmarks, its efficacy is questioned due to its limited detection capabilities. In addition, due to radiation health risks, only short static analyses were applied.

Magnetic resonance imaging (MRI) could provide valuable and accurate information for determining the integrity of the skeleton and soft tissues in a transfemoral stump [9]. Comparatively, even though state-of-the-art X-ray spiral computed tomography (SXCT) provides very detailed images, they are still inferior to MRI images. In addition, there are no known side effects with MRI, while the use of SXCT requires ionising agents. However, MRI is very costly to use, and with its relatively long scanning time compared with computed tomography (CT) and ultrasound, results in limitations for extensive clinical use.

Thus, imaging of the residual limb while enclosed within a socket has to be determined by another method. Ultrasound was chosen as it is relatively inexpensive, requires a short scanning time, and has no known ill effects. It is primarily used to evaluate soft tissue geometry, since high attenuation occurred between bone and its surroundings. Still, there has been increasing use of ultrasound for building 3D images of the residual limb [10–12].

Murray and Convery (2000) have previously investigated the feasibility of using ultrasound sensors to monitor the dynamics of the femur within a socket [13]. Accuracy and repeatability tests performed in a water-filled socket demonstrated that it was possible to obtain useful measures of femoral motion during gait. They also reported that the femur performed an extension of 6° and an abduction up to 9° by midstance, and then performed a flexion of 6° and an adduction of 2° by toe-off during level walking [14]. However, the difficulty in mounting the transducers and the time-consuming analysis of the ultrasound images, meant that this could not be adapted for extensive clinical use.

To the best of the authors’ knowledge, none of the stated methods have been extensively applied in clinical prosthetics. Traditionally, the quality of a socket fitting is subjective as it relies heavily on the observation of the prosthetists and feedback from the amputees. Questionnaires can also be used to assess outcomes from the use of lower limb prostheses [15]. While feedback from the amputees plays an important part in determining the level of comfort and functionality of a prosthesis, there is also a need to develop transducers to quantify socket fitting. Currently, most of the instrumentation used during socket fitting focuses on the external measurements of the stump or at the stump/socket interface such as pressure distribution and gait parameters. Investigation into the internal geometry of the stump has been limited due to associated health risks and costs from traditional imaging techniques such as X-rays and CT.

Therefore, we wanted to explore the use of ultrasound in determining the position of the femur in a residual limb. This study aims to gauge the effectiveness of using an A-mode ultrasound system i.e. where we receive amplitude versus time data. This allows the use of light-weight ultrasound transducers, rather than the conventional (and bulky) mechanical probe. Here, we determined possible measurement errors from the use of our equipment, so as to better gauge its feasibility when translated to prosthetic use during clinical trials.

## Methodology

This study aims to calculate and report the measurement errors when one or two ultrasound transducers are utilized in a controlled environment, such as a water bath. We determined errors related to distance, material thickness, orientation and reproducibility. We further applied the use of this ultrasound system to two subjects; one being a normal subject, and the other being a transfemoral amputee.

### Equipment

**Fig 2:**
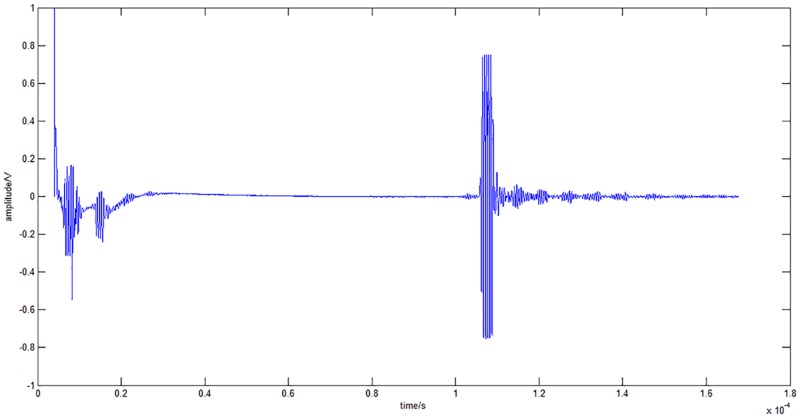
A typical ultrasonic waveform (detecting position of a rod in a water bath).

The PCM 8 Channel 100 MHz Ultrasound platform system from Inoson Gmbh (St. Ingbert, Germany) was selected for this study. The 2 MHz transducer weighs 4 g (Fig 1). Descriptions are given in Table 1. Sound field calculations are provided in S1 File. Ultrasound gel Echoson-Ultraschallgel GU 401 (Sonogel, Bad Camberg, Germany) was chosen as it possessed similar acoustic impedance. The received signals from this ultrasound system were recorded as a waveform trace (Fig 2).

**Table 1:** Description of ultrasound transducer used in this study.

Type	Contact
Nominal frequency	2 MHz
rel. 6 dB transmission bandwidth	50%
Aperture	2 elements 8 mm x 4 mm
Resonator	1–3 Piezocomposite
Physical dimensions	⌀18 mm x 8 mm
Connection	2 micro-coaxial cable 50 mm with MMCX-connector
Environmental conditions/ applications	Water (without chemical additives) or solids
Working temperature	10°C– 40°C
Maximum immersion depth	1 m
Immersion time	Max 1 hr
Max transmitting voltage	100 Vpp at 1:1000 duty cycle

### Distance and material thickness

**Fig 3:**
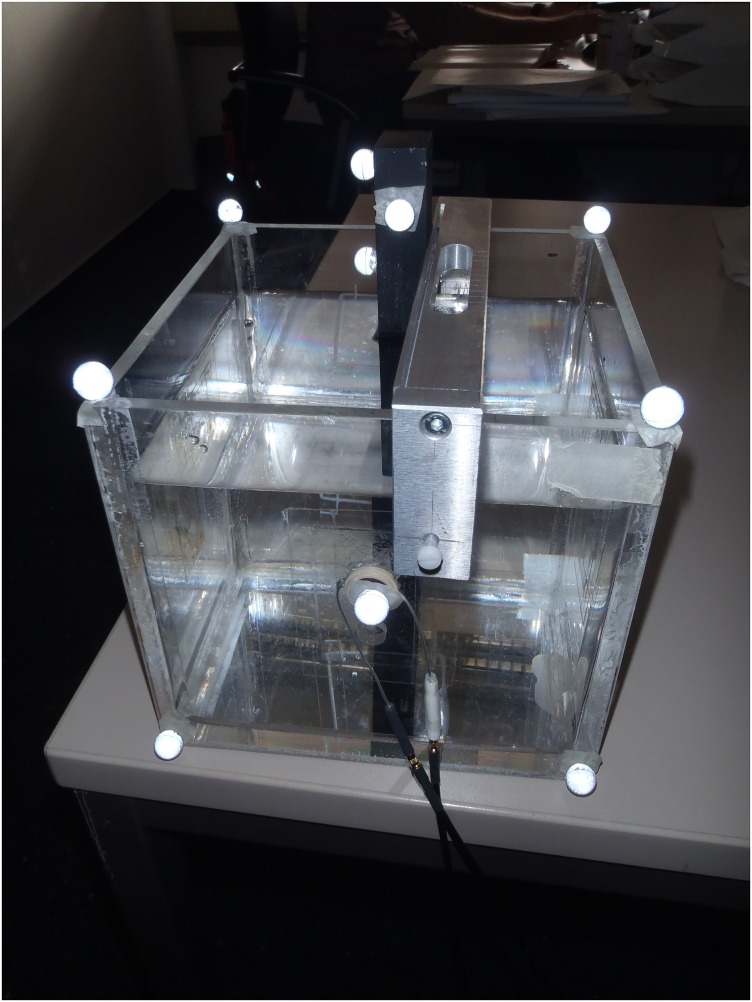
A double-material rod, made of steel and aluminium metals, in a water bath with reflective markers.

Ultrasound measurements were performed to determine the random movement of a rod in a water bath measuring 16.0 cm x 16.0 cm x 16.0 cm (Fig 3). Water temperature was measured to be at 25.7°C at the beginning of the tests.

**Fig 4:**
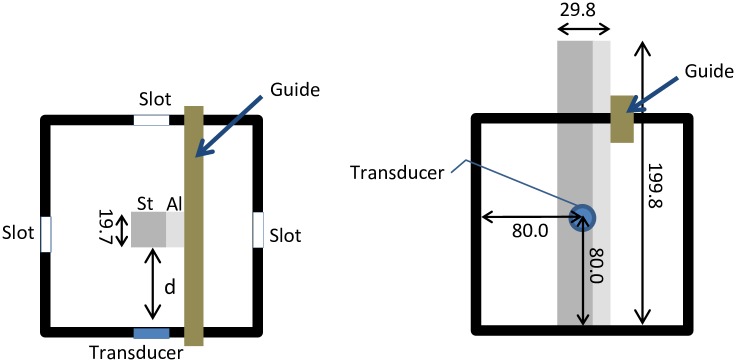
A double-material rod, made of steel (St) and aluminium (Al) metals in a water bath (left—top view, right—side view). All measurements are in mm.

One small slot was created in each of the four vertical walls of the water bath to accommodate a transducer. The slot was determined to be right in the middle of the wall; 80 mm from each side (Fig 4). A thin polyethylene sheet was attached over the slot such that the sheet comes between the transducer and water. The polyethylene sheet, thus, acts like a seal to prevent water from seeping out of the water tank, and also simulates ultrasound measurements over skin. The rod measured 19.7 mm x 29.8 mm x 199.8 mm in total; It was made of a 9.8 mm thick aluminium alloy (Al) and a 19.7 mm thick non-alloy quality steel (St) attached together with 0.3 mm thick Pattex Ultra Gel (Düsseldorf, Germany). This was used to determine if the ultrasound could differentiate between adjoining materials.

The rod was randomly placed in the water bath at random distances *d* directly in the path of the ultrasound beam, and steered by a guide (Figs 3 and 4). Then, the distance between the edge of the rod facing the ultrasound transducer, and the thickness of the metals were determined. A total of 18 trials were performed: six trials were performed with the thinner Al facing the transducer; six trials were performed with the thicker St facing the transducer; six trials were performed when both materials were facing the transducer (Fig 4). Processed signals from the ultrasound equipment were compared with measurements using two 3D motion capture cameras from Qualisys (Gothenburg, Sweden) whose accuracy is well known [16]. As depicted in Fig 3, six reflective markers of 10 mm in diameter were attached to the water bath, while three reflective markers were placed on the rod.

### Orientation

**Fig 5:**
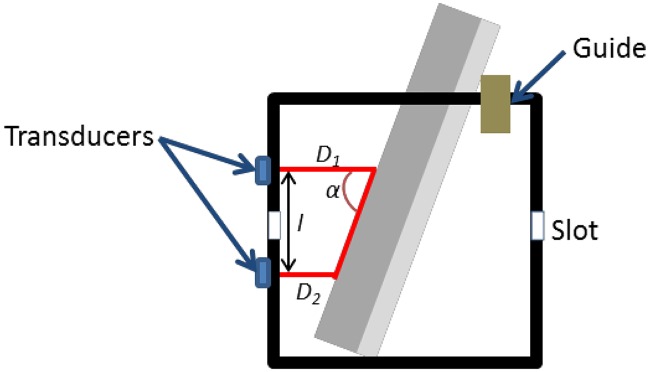
A rod rotated in a water bath, and supported by a guide (side view).

**Fig 6:**
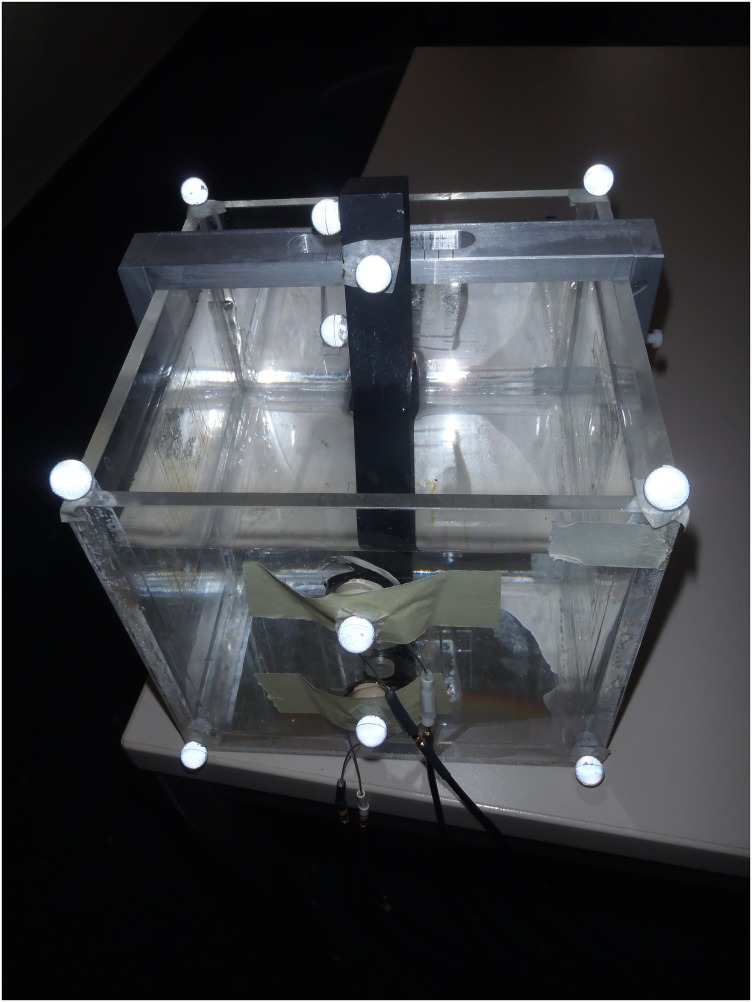
A rod rotated in a water bath with reflective markers.

While the use of a single transducer was sufficient to detect the position of a rod, it is insufficient for determining the orientation of the rod. For this, one needs a minimum of two transducers vertically positioned at the same wall. In our study, two transducers were placed above and below the slot (Fig 5). Qualisys motion capture system calculated a distance of *l* = 63.2 mm from reflective markers placed on the transducers (Figs 5 and 6). The angular position of the rod was randomly adjusted in the water bath, and supported by a guide. A total of 18 trials were performed. *D*_*1*_ and *D*_*2*_ were determined via the ultrasound system (Fig 5). Thereafter, angle of rotation α is calculated as:

$$\text{tan}\left(\propto \right)=\frac{l}{D_{1}-D_{2}}$$

Results calculated from ultrasound were then compared with angles determined by the Qualisys system.

### Reproducibility tests

**Fig 7:**
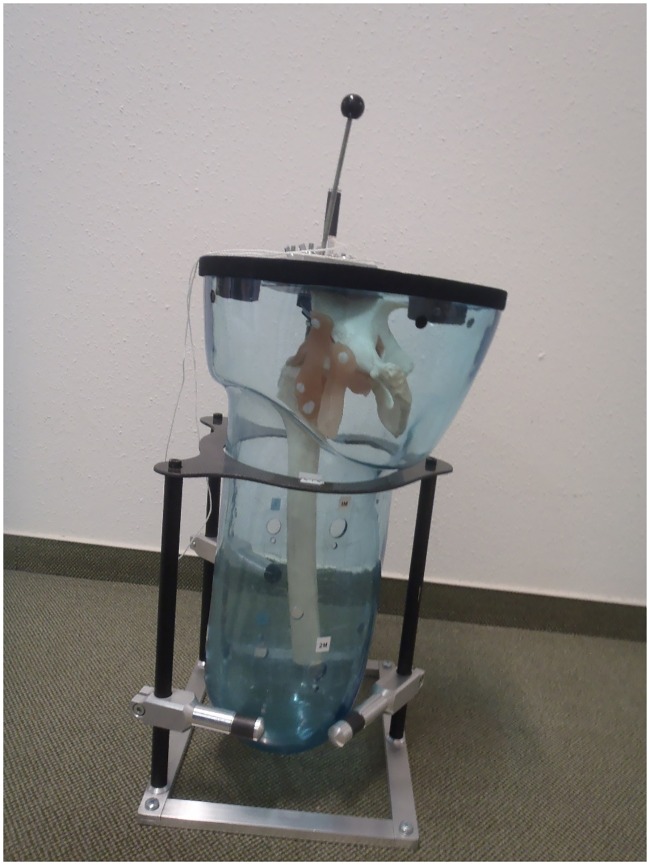
Replica of a stump.

**Fig 8:**
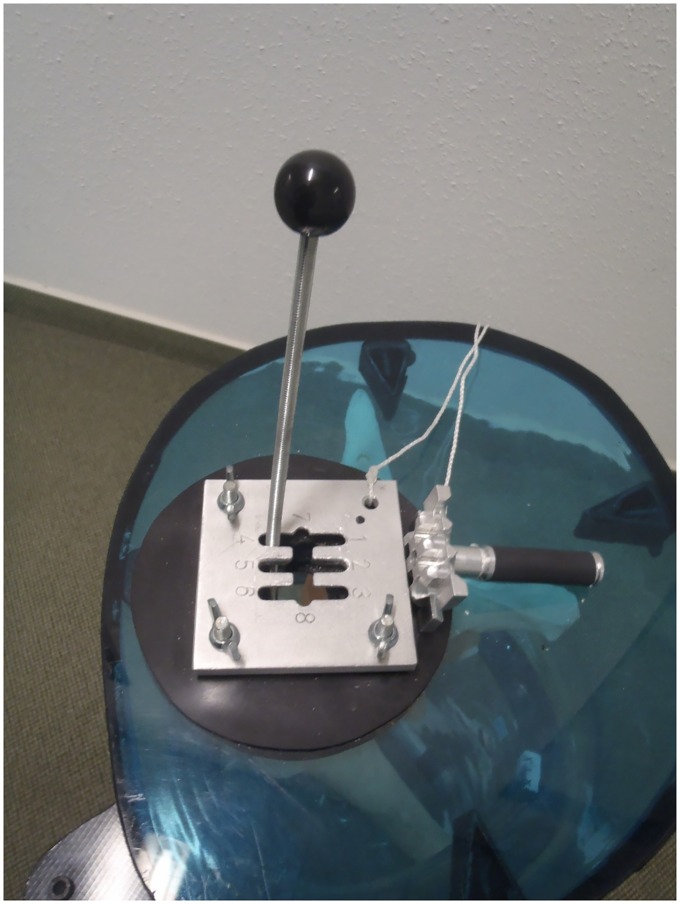
Gear lever for moving the femur to different positions, and a stopper to keep the femur in place at each gear.

**Fig 9:**
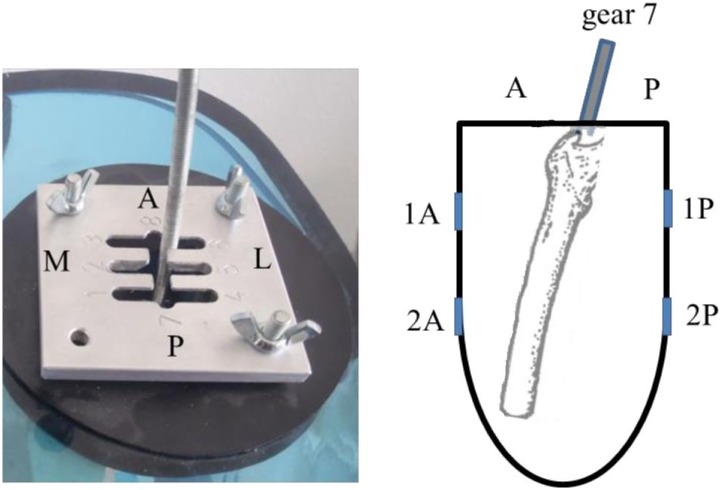
Close-up of gear lever. Note that if lever is moved towards the posterior side of the stump cast (gear number 7 at the ‘P’ side), the residual femur is actually moved nearer the anterior side of the stump cast. ‘M’–medial, ‘L’–lateral, ‘A’–anterior, ‘P’–posterior.

**Fig 10:**
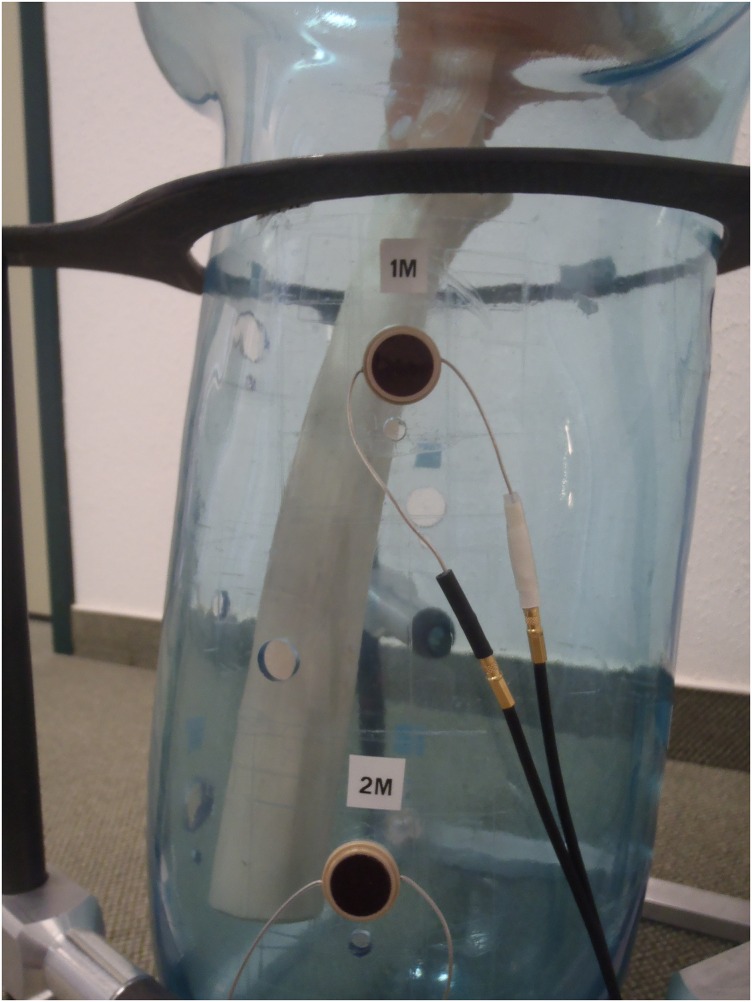
Two slots for transducer attachment at the medial side (1M, 2M) of the stump cast.

Signals received are sensitive to small adjustments during transducer placement. Ideally, the transducer should be placed perpendicular to the bone such that the signal received is at its maximum strength [17], but this is difficult to determine in clinical studies. Reproducibility is the closeness of the agreement between measurements performed in different sessions. The reproducibility of the ultrasound measurements was determined by measuring the position of a plastic residual femur submerged in water, within a cast shaped like a stump (Fig 7). A gear lever was mounted on top of the cast such that the femur could statically simulate different flexion/extension and ab/adduction angles. A stopper ensured that the femur would be held in place at the same position at each gear (Figs 8 and 9). Thus, the position of the femur could be reproduced at different times just by simply shifting the gear lever. The stump cast was fitted with eight slots for the attachment of the ultrasound transducers. Two transducers can be placed at each plane: anterior, posterior, medial and lateral (Fig 10). This allowed the ultrasound transducers to determine distance of the femur within the cast in all four planes. In our study, several measurements were recorded from the same location. After each measurement, the transducer was removed, and then replaced later at the same slot. The tests were performed a total of five times by the same researcher. Water temperature was measured to be at 25.7°C at the beginning of the tests.

### Subjects

We further applied this study in-vivo. In a water bath, the ultrasound beam passed through a homogeneous material such as water, allowing a clear signal, such as the one shown in Fig 2, to be recorded. When performed on human subjects, the signals received would suffer from attenuation. Furthermore, the ultrasound beam would encounter a number of interfaces (such as fat-muscle) before it reaches bone. Bone positions calculated from MRI data were compared with ultrasound measurements. Informed consent, both written and verbal, was obtained from all subjects before their participation in the study. Ethics approval was obtained from the University Hospital Tübingen (project nr: 587/2014BO1).

**Fig 11:**
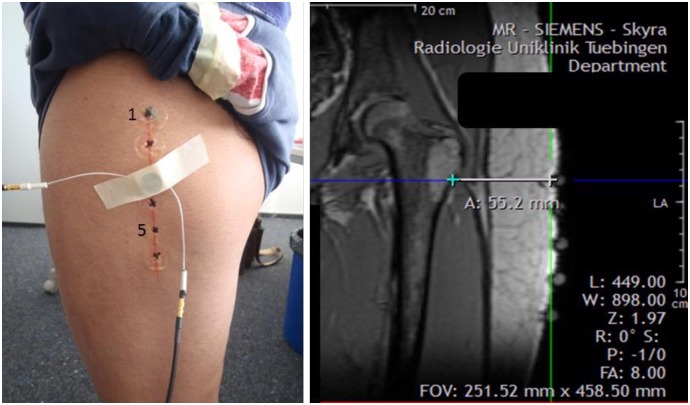
Ultrasound transducer placed on one of the marked positions on subject #1 (left). MRI of left limb of subject #1 with visible MRI markers (frontal plane) (right).

Subject #1 is a normal healthy female who volunteered for this study. She is 28 years old, 1.53 m tall and weighs 56 kg. Five positions, two cm apart, were marked along the femur (Fig 11). A single 2 MHz transducer was utilized each time. Three trials were performed, in which the transducer was removed and re-placed at the same marked position by the same researcher. Positions of the ultrasound transducers on the thigh were marked, and replaced at the same locations by magnetic resonance imaging (MRI) markers. A MRI scan was performed with a 3T whole-body MRI scanner (Magnetom Trio, Siemens Healthcare, Erlangen, Germany).

**Fig 12:**
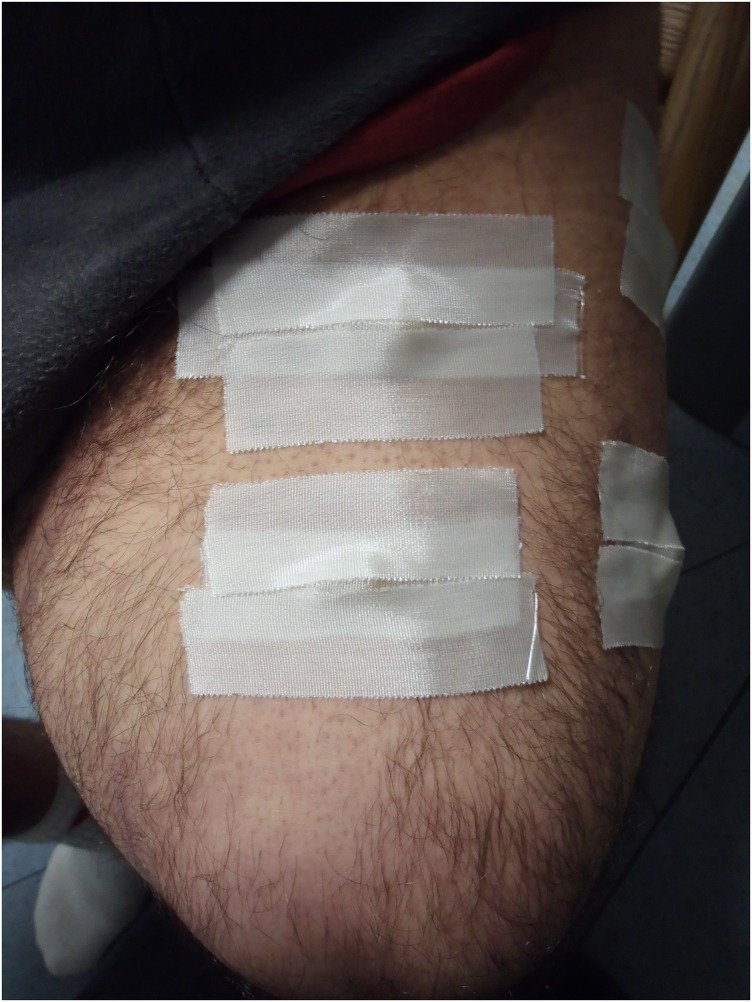
MRI markers correlating with locations of the ultrasound transducers on the stump.

Subject #2 is a 44-year-old male whose left limb was amputated above the knee one year ago as a result of an accident. He is 1.67 m tall and weighs 76.7 kg with the prosthetic leg. The stump length measured approximately 29.5 cm from the trochanter, with a maximum circumference of about 52.0 cm. Four 2 MHz ultrasound transducers were placed on his stump. Placing the transducers over the prosthetic socket yielded no results. Since the femur is most distal, lateral and posterior during full weight-bearing on the prosthesis [8], we placed two transducers each at the anterior and lateral planes of the stump. Ultrasound measurements were not performed at the posterior positions of the stump as the MRI markers were displaced during the MRI scans. Ultrasound measurements were performed during quiet standing without wearing the prosthetic limb and the subject holding on to a support. This position would be similar to the stump position during midstance, but without loading. Positions of the ultrasound transducers on the stump were marked, and replaced at the same locations with MRI markers (Fig 12). A MRI scan was performed with a 3T whole-body MRI scanner (Magnetom Trio, Siemens Healthcare, Erlangen, Germany). Bone positions calculated from MRI data were compared with the ultrasound measurements.

### Data processing

**Fig 13:**
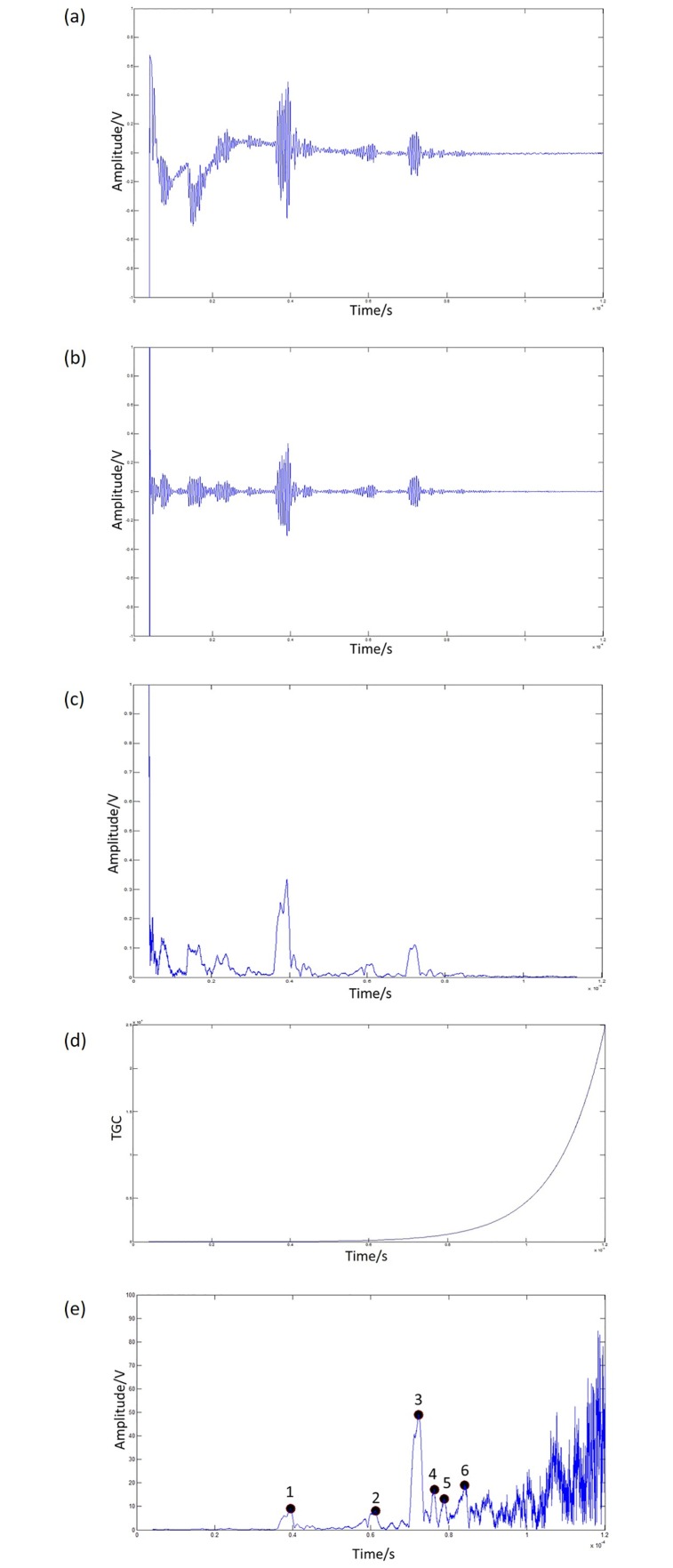
Analysing ultrasound pulse-echo measurements performed on human subjects: (a) raw signal, (b) band-pass filtered data, (c) real and absolute values of the Hilbert-transformed data, (d) time gain compensation (e) interested peaks (marked circles).

The raw signal was first processed using a band-pass filter with a low cut-off frequency of 60000 Hz and a high cut-off frequency of 1.5 MHz (Fig 13b). The filtered signal then underwent a Hilbert transform [18], which provided the analytical envelope of the ultrasound data (Fig 13c).

Since the signal decreases exponentially with penetration depth, a time gain compensation (TGC) was introduced to compensate for this attenuation (Fig 13d). The TGC equation used is a reverse exponential function, since the signals decreases exponentially.

$$I(t)=I_{o}(t)e^{2ar}$$

where *I*_*o*_ is the signal at time *t*, *a* is the attenuation coefficient (in dB/cm) and *r* is the location at time *t* (in cm). We used *a* = 0.0076 dB/cm for water at 25°C for 2 MHz [19]. The attenuation of water is very small, and so the TGC used in the water bath may be of very little significance. However, the attenuation in soft tissue can be significant. We used *a* = 0.54 dB/MHz.cm to define the average attenuation for soft tissue [20]. From the amplified Hilbert-transformed data, signal onset is taken as the time when a peak occurred (marked circles in Fig 13e).

Thus, *d*, which is the distance of the rod from the transducer, can be calculated as:

$$d=\frac{s*t}{2}$$

where *s* is the speed of sound in medium, and *t* is the time taken for the echo signal to return to the transducer. The speed of sound in water at 25°C is taken as 1497 m/s. The speed of sound for aluminium and steel is 6400 m/s and 5960 m/s respectively.

For the ultrasound measurements performed in-vivo, an echo signal in the form of a peak is detected every time it passes an interface, where soft tissues of different densities occur, such as a fat-muscle interface. The ultrasound beam would encounter a number of interfaces before it reaches bone, and so we expected a number of peaks. A peak is identified if it is greater than a pre-determined threshold. Mozes et al., (2010) identified a peak as greater than 1.5 times the mean of the entire signal. In our study, a threshold was first determined from the first 5000 frames (or the first 5.4x10^-5^ s of data) [17]. A peak is identified if it is above three times this threshold, and more than three times the threshold compared to the surrounding data (Fig 13e). A magnitude three times the threshold was chosen in order to be more selective in detecting a peak of interest, compared to a lower threshold. From the time it takes for the peaks to be detected, we can determine the distance *d* from the transducer to the soft tissue interface. Here, the average speed of sound for soft tissue is taken to be 1561 m/s [20].

We first made the assumption that the muscle-bone interface would not be more than six interfaces away from the skin. Therefore, the maximum peak from the first six identified peaks was picked. Fig 13e shows an example of the third peak being picked. Referring to the MRI (Fig 11), we think the peaks may correspond to points where differences in soft tissue densities occurred.

## Results

**Fig 14:**
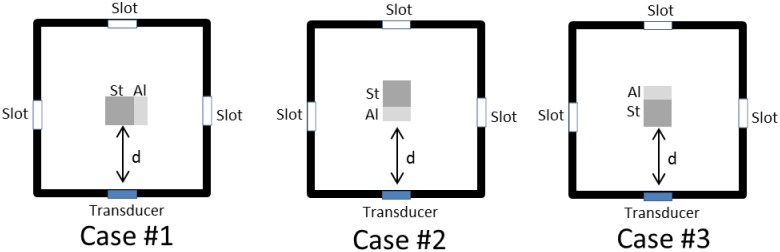
Calculated distance *d* for cases #1 to #3 (Table 2). The rod was made of aluminium (Al) and steel (St).

**Fig 15:**
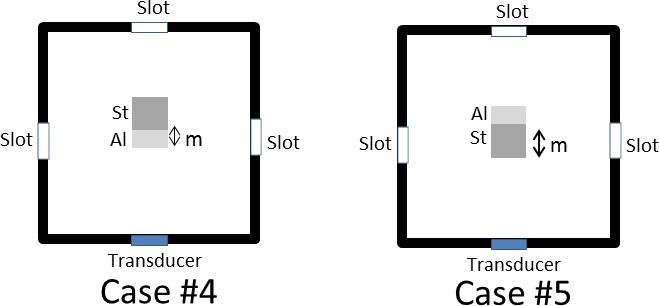
Calculated material thickness *m* for cases #4 and #5 (Table 3). The rod was made of aluminium (Al) and steel (St).

The rod was randomly placed in the water bath at distances ranging from 14.6 mm to 112.6 mm from the ultrasound transducer. Errors calculated by the ultrasound were found to be within 2.9 mm to 8.4 mm. The mean total error in the calculated distance is 5.2 mm ± 2.5 mm (Fig 14, Table 2). The mean total error in determining the material thickness is 2.5 mm ± 2.1 mm (Fig 15, Table 3).

**Table 2:** Mean difference (diff) and standard deviation (std) of calculated distance *d* between ultrasound and Qualisys measurements (in mm).

	Case #1	Case #2	Case #3	Mean diff
	n = 6	n = 6	n = 6	n = 18
diff	8.4	4.4	2.9	5.2
std	0.5	0.4	1.3	2.5

**Table 3:** Mean difference (diff) and standard deviation (std) in the calculated material thickness *m* (in mm).

	Case #4	Case #5	Mean diff
	n = 6	n = 6	n = 12
diff	0.9	4.1	2.5
std	0.6	1.8	2.1

The rod was randomly placed at different angles during the orientation test. Angles ranged from 70.8 degrees to 90.0 degrees, and errors calculated were found to be 1.2 degrees ± 1.3 degrees.

For the reproducibility tests, the maximum standard deviation of all readings is 3.9 mm at gear 4, indicating that the ultrasound system can provide measurements that have a high level of agreement when performed at the same location. Full results are reported in S1 Table. Note that if the lever is moved towards the posterior wall of the stump cast (for example, gear 7), the residual femur is actually moved nearer to the anterior wall of the stump cast (Fig 9). So, when an ultrasound transducer was placed at 1P (the proximal posterior position), the distance of the femur from the anterior wall is shorter than the posterior wall.

**Fig 16:**
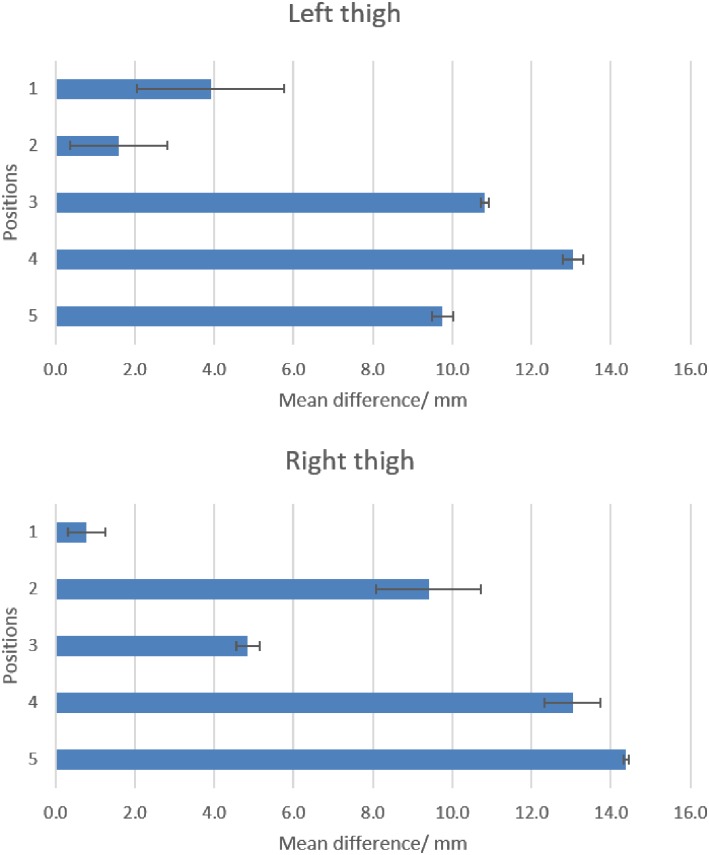
Mean difference (diff) and standard deviation of the femur from the thigh surface between MRI scans and ultrasound measurements in Subject #1. Positions on thigh shown in Fig 11. All measurements are in mm.

**Fig 17:**
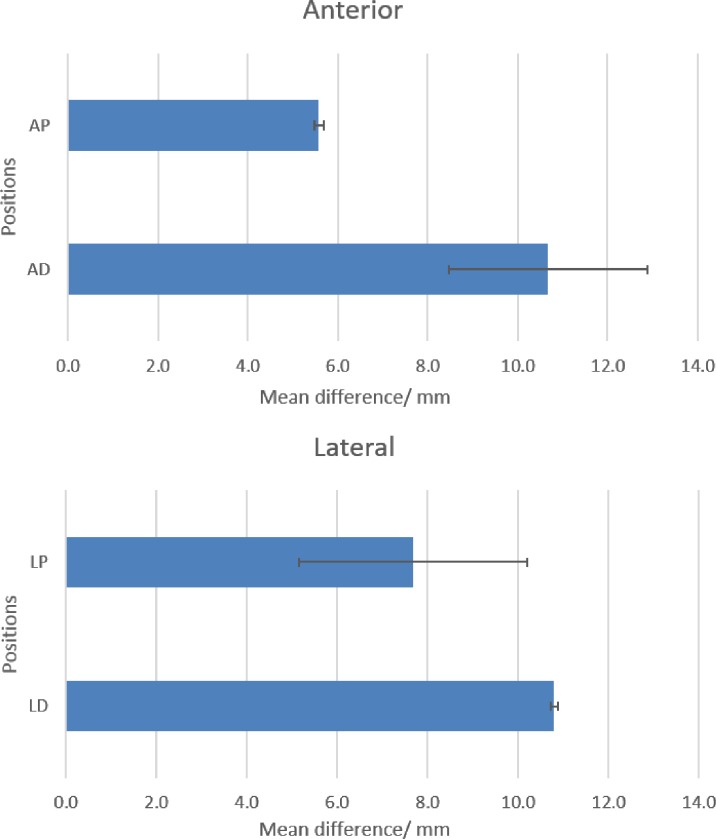
Mean difference (diff) and standard deviation of left residual femur from thigh surface between MRI scans and ultrasound measurements in Subject #2. ‘P’ denotes proximal placement, ‘D’ denotes distal placement. ‘L’–lateral, ‘A’–anterior. All measurements are in mm.

While the position of the ultrasound transducer was known, we were uncertain of the direction or path of the ultrasound beam. From the MRI scan for subject #1, we made an approximation of the bone distance within the thigh from the frontal plane (Fig 11). From the MRI scan for subject #2, the distance of the residual femur from the anterior and lateral MRI markers were determined from the sagittal and frontal plane respectively. To minimise human error, the distances in the scans were determined by the same researcher. Errors of 0.5 mm to 14.4 mm were found for subject #1 (Fig 16). Errors of 5.6 mm to 10.8 mm were found for subject #2 (Fig 17).

## Discussion

Attempting to determine the position of the femur within a stump using ultrasound is not new. Ultrasound has been suggested as a safe and cheap alternative to X-rays or MRI methods, and has been utilized in previous studies to monitor the motion of the femur. Convery and Murray (2000) demonstrated that ultrasound B-scans can provide information about the residual bone within a socket during gait [14]. However, there were challenges in adapting their method for application in clinical prosthetics. The current impact of A-mode ultrasound measurements in lower limb prosthetics is limited. In this study, we present an estimate of the errors calculated with the use of an A-mode ultrasound system.

In the experiments performed in the water bath, accuracy was good as the ultrasound beam passed through homogeneous materials such as water, aluminium or steel. A clear ultrasound signal could be obtained in a water bath (as shown in Fig 2). A single ultrasound transducer was sufficient to detect the position of a double-material rod in the water bath with an error in the range from 2.9 mm to 8.4 mm (Table 2). Errors are reported to be higher when both materials of the rod faced the transducer. This demonstrates that a non-homogenous interface would affect the accuracy. Using ultrasound, we can detect the interface of the adjoining metals with an approximate error of 0.8 mm to 4.1 mm (Table 3). Furthermore, the attenuation coefficient of water is relatively low [19]. So, our equipment setup in utilizing an A-mode ultrasound system, can be applied to determine the position and material thickness of a rod within a water bath.

The mean total error in determining the material thickness is 2.5 mm ± 2.1 mm (Table 3), which is approximately a 2% to 25% error. This is much higher than the results reported by Metwally et al., (2016), who reported a mean error of less than 1.5% when they determined the thickness of animal cortical bone and wood samples [21]. We interpreted our signals according to the amount of time ultrasound takes to encounter materials of different densities, whereas Metwally et al., (2016) analysed their measurements using a wavelet-based processing method, which may be more consistent than ours [21].

Errors calculated during the orientation tests were found to be approximately 1.2 degrees ± 1.3 degrees. This is relatively higher than Convery and Murray (2000), who reported an error of <1 degree when they performed their study using a B-mode imaging system [14]. In this instance, we are uncertain if the larger errors were due to the equipment or data processing method. Shen et al., (2014) developed a metamaterial that allowed three times as much ultrasound waves to pass through bone. This, however, is still in the experimental stages [22]. A better system that could minimise attenuation in soft tissues would invariably contribute to greater accuracy.

Larger errors are expected in human subjects; possible contributors are subject motion during recording and signal attenuation from soft tissues. Also, although we performed our measurements at the unloaded midstance position that is similar to the supine scanning position in an MRI, we noticed that the external markers at the stump posterior were compressed during the MRI scans. As such, we did not perform any comparisons at the posterior side of the stump, as we are aware that the shape of the stump may be altered while lying down. Compared to the measurements of femur location as determined by MRI, results showed that differences as much as 14.4 mm were reported for ultrasound measurements (Figs 16 and 17). This is almost twice the maximum error of 8.4 mm found for tests done in the water bath. In a normal subject, the orientation of the femur can be estimated to lie between the trochanter and the lateral condyle. However, this is more difficult to gauge in an amputee. Also, while we think that placing the transducer perpendicular to the thigh surface would yield more accurate results, this is, however, difficult to perform during in-vivo experiments.

When transducers were placed on the stump, errors of up to 14.4 mm were reported (Fig 16) during in-vivo experiments. Placing the transducers over the prosthetic socket yielded no results. It is likely that an air gap exists between the stump, liner or socket, since most of the ultrasound signal that passes through an air gap would be reflected. To circumvent this problem, Convery and Murray (2000) fabricated a duplicate socket to house an ultrasound transducer so as to enable good contact with the stump [14]. Likewise, we could also incorporate the transducers in a socket. However, this will not be practical for clinical use.

Our study has limitations particularly during in-vivo measurements. Only one amputee subject was utilized, and measurements were performed at only four positions on the stump. Therefore, the data presented here should only be considered as preliminary data. Additionally, our current results are only based on measurements of static positions. We hope to mitigate this problem by configuring our system to record measurements dynamically.

Following these measurements, we hope to develop the use of A-mode ultrasound for long- term and extensive use in clinical studies. We also hope that the application of ultrasound during socket fitting will become a standard feature, if technical difficulties can be reduced. There are still several challenges we may face until this technology can be translated to clinical practice, namely ensuring that the femur does not move out of the field of view of the ultrasound beam, setting up a detailed analysis of sensor placement so as to fully capture the femur, and robust data analysis during dynamic measurements. So, one of our future aims regarding the field of view is to investigate the use of multiple transducers around the stump during in-vivo measurements. Still, the advantages with the use of this ultrasound equipment, such as its low cost, light weight transducers, portability and ease of use, may play a huge part in the biomechanical understanding of the internal dynamics within the stump, and is thus, worth exploring.

## Supporting Information

S1 File Supporting file. (PDF)

S1 Table Supporting table. (PDF)
