# Supplementary material for: Exploring the Use of Non-Image-Based Ultrasound to Detect the Position of the Residual Femur within a Stump
Source: PLoS One. 2016 Oct 20;11(10):e0164583. doi: 10.1371/journal.pone.0164583 (PMC5072695; doi:10.1371/journal.pone.0164583)
Supplement: S1 File — (PDF) [file pone.0164583.s001.pdf]

S1

Pulse response/spectrum

Method	Pulse/ Echo
Gap/ interval	10 mm
Direction	Coplanar sheet
Medium	Plexiglas, 20 °C
Transmission signal	Pulse

Element 1

Amplitude/ V

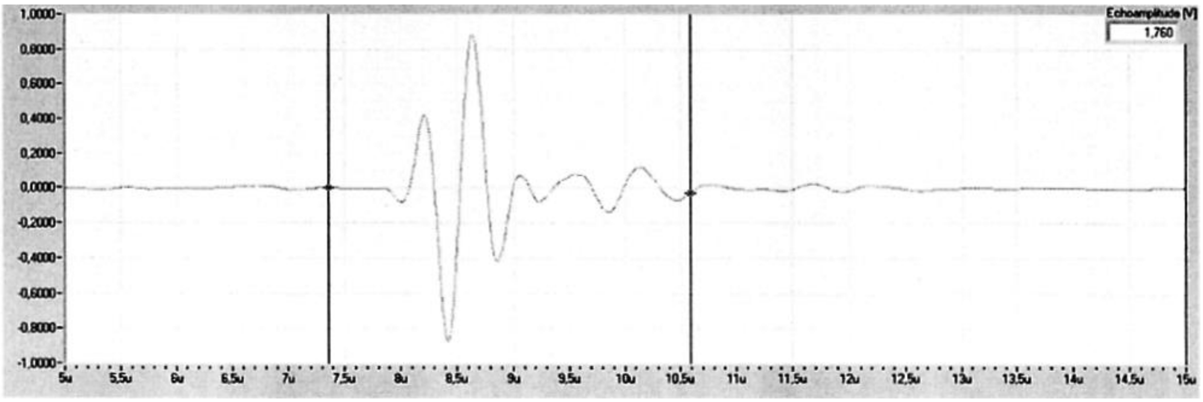

time/ s

Element 2

Amplitude/ V

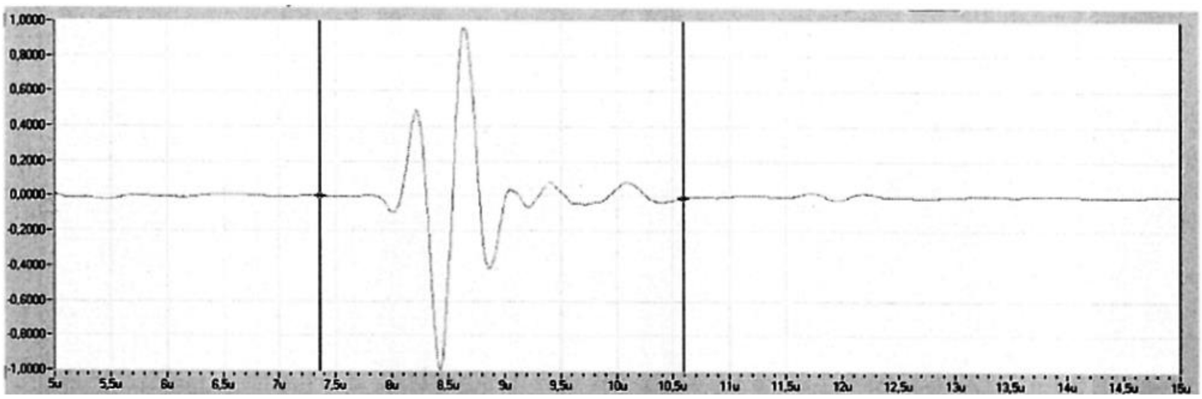

time/ s

Burst operation/ Sensitivity

Method	Pulse/ Echo
Gap/ interval	10 mm
Direction	Coplanar sheet
Medium	Plexiglas, 20 °C
Transmission signal	Burst 2 MHz/ N=5

Element 1

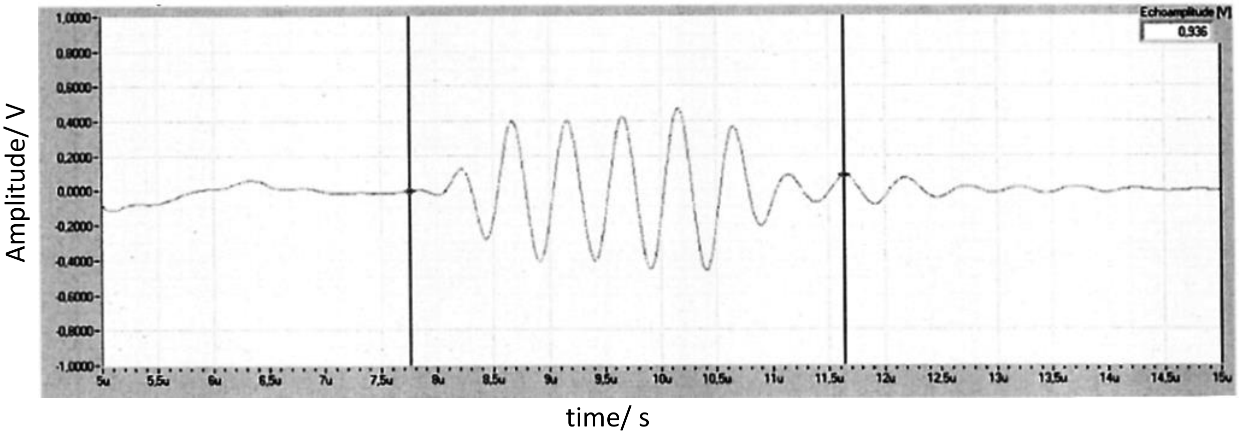

Element 2

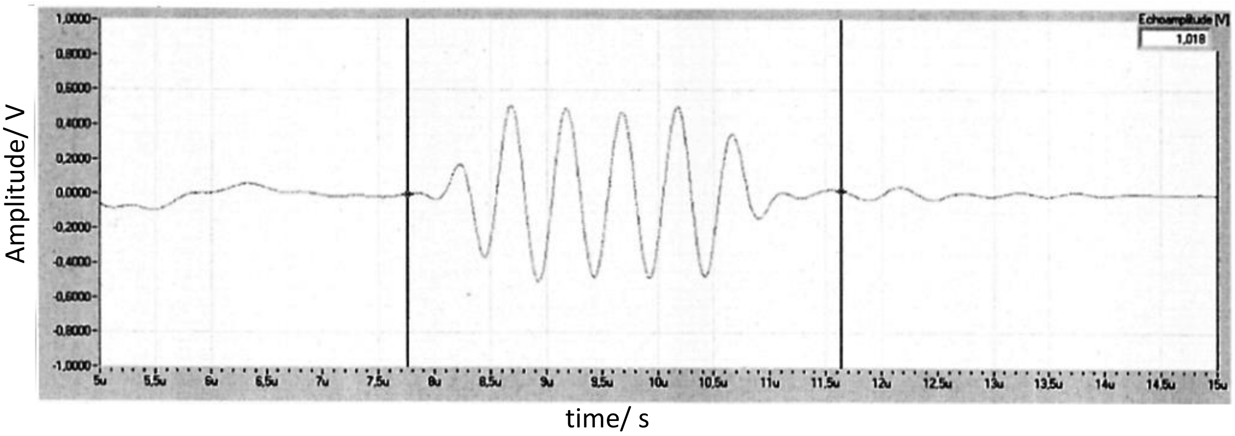

Analytical sound field calculations for rectangular pistons

Protocol

Speed of sound of medium	c = 1.5 x 10^6
Centre frequency	f = 2 x 10^6
Cross section dimensions	D1 = 8 mm<br>D2 = 4 mm
Near field length	N = 8.146 x 10^{-3}
Lateral 6 dB-width	FWHM = 1.222 x 10^{-3}

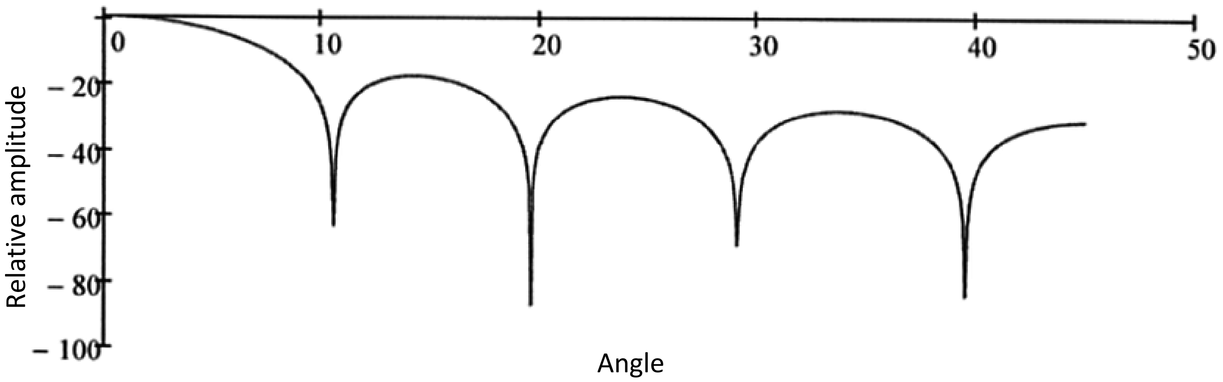

Full 3 dB-width	dB3 = 9.018 / 6.103
Full 6 dB-width	dB6 = 12.265 / 8.246

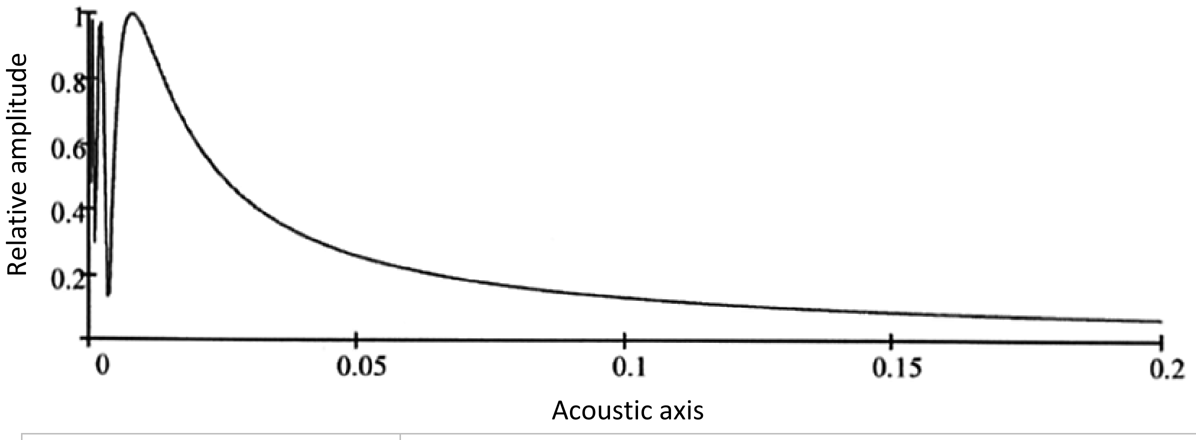

Full 3 dB-width	w3dB = 0.012
Full 6 dB-width	w6dB = 0.021
