# Supplementary material for: Exploring the Use of Non-Image-Based Ultrasound to Detect the Position of the Residual Femur within a Stump
Source: PLoS One. 2016 Oct 20;11(10):e0164583. doi: 10.1371/journal.pone.0164583 (PMC5072695; doi:10.1371/journal.pone.0164583)
Supplement: S1 Table — (PDF) [file pone.0164583.s002.pdf]

Gear	1	2	3	4	5	6	7	8
**1L**	NB	35.9±0.0	39.7±0.5	NB	77.9±0.0	72.4±0.7	NB	60.0±1.6
**2L**	NB	NB	NB	NB	NB	NB	NB	NB
**1M**	94.5±0.5	NB	NB	58.7±0.3	64.1±1.6	NB	78.3±1.7	70.1±2.0
**2M**	94.5±0.1	75.6±0.8	NB	NB	22.7±0.6	NB	NB	NB
**1P**	88.7±1.4	78.9±0.3	68.6±1.1	88.5±3.9	86.0±2.8	77.5±1.2	87.5±0.0	65.8±1.0
**2P**	95.0±1.8	77.5±1.0	42.8±0.2	NB	NB	NB	NB	NB
**1A**	NB	53.6±0.4	NB	46.4±0.3	53.2±2.1	56.8±0.0	37.4±0.4	61.3±0.1
**2A**	NB	NB	NB	NB	NB	NB	25.7±0.7	81.4±0.1

S2 Table. Mean and standard deviation values from reproduced ultrasound measurements for eight different gear positions. ‘1’ denotes proximal, ‘2’ denotes distal slot at each plane. ‘M’ – medial, ‘L’ – lateral, ‘A’ – anterior, ‘P’ – posterior. ‘NB’ denotes no bone detected. All readings in mm.
